# The assessment of image quality and diagnostic value in X-ray images: a survey on radiographers’ reasons for rejecting images

**DOI:** 10.1186/s13244-022-01169-9

**Published:** 2022-03-04

**Authors:** Elin Kjelle, Catherine Chilanga

**Affiliations:** Faculty of Health and Social Sciences, Department of Optometry, Radiography and Lighting Design, University College of Southeast Norway, Notodden, Norway

**Keywords:** Radiography, Radiology, Retake, Image quality, Survey

## Abstract

**Background:**

Assessing the quality of diagnostic images is subjective and influenced by factors such education, skills, and experience of the assessor. This study aims to explore the radiographers’ assessments of medical usefulness or rejection of X-ray images in specific cases.

**Results:**

Eighty-one radiographers from different countries responded to the questionnaire distributed online at the EFRS research HUB at ECR 2020 (a 15% response rate). Forty-two percent of the respondents practiced in the UK and Ireland. In addition to rejecting or keeping images in the presented 30 cases and giving a main reason for the images rejected, the participants explained their choice using comments, 1176 comments were obtained. Sixty percent of the comments were on kept images. The respondents kept on average 63% of the images. In the “Keep”, “Could keep”, and “Reject” categories on average 84%, 63% and 43% of images were kept respectively. The most common reasons given for rejecting an image were suboptimal positioning and centering. Potential diagnostic value and radiation protection were indicated as reasons to keep an image perceived as of low quality reported in *n* = 353 and *n* = 33 comments respectively.

**Conclusions:**

There is an agreement internationally on what makes a good quality X-ray image. However, the opinion on medical usefulness of images of low or poor quality compared to image criteria varies. Diagnostic capability and radiation protection was the rationale used for keeping images not fulfilling image criteria. There seems to be a need for diagnostic quality to be included in image assessment in clinical practice.

## Key points

- Radiographers internationally highly agree on what makes a good X-ray image.
- Assessing images mainly against image criteria may lead to more rejections.
- Training for radiographers in image quality assessment and regular reject analysis might reduce retake rate.
- Dialogue between radiologists and radiographers on diagnostic image quality is important in clinical practice.

## Introduction

The quality of a radiographic image influences diagnostic accuracy and subsequent clinical management of the patient [1]. A radiographic image is accepted as good quality when certain technical qualities are satisfied, and the image considered of diagnostic value [2]. An image of low quality has the possibility of rejection and the radiological procedure repeated in attempt to produce imaging of diagnostic value [3]. Rejects and subsequent retakes of radiographic images expose both patients and radiological personnel to unnecessary ionising radiation [4]. Over exposure to ionising radiation increases risk of probability of radiation induced stochastic effects [5]. Repeated imaging further results to higher usage of both human and radiological resources [6]. Jones et al. [3] state that rejected images are integral to X-ray imaging, where patient positioning and alignment are vital components of image quality. During a radiological procedure, patient motion, positioning, and artefacts unique to the image receptor technology can result in repeated images [3] and are reported as the major reasons for image retakes [7]. Examining the underlying causes for image rejections assists to identify any technical or training issues required in a radiology department [8]. This further align with the As Low As Reasonably Achievable, (ALARA) principle and global initiatives of imaging wisely for proper use of radiological resources [3, 9].

Image quality assessment is reported to be subjective and significantly differs between and among radiographers and radiologists. Dunn and Rogers [10] found that radiologists retained as useful 50% more of the images that showed positioning errors compared to radiographers, indicating a difference in clinical judgement between the two groups. Kjelle et al. [11] further report that although there is a professional agreement on the assessment of good quality images, radiologists tend to accept a higher number of images that radiographers reject and considered to be of low quality. Mount [12] suggests that radiographers generally tend to favour technical characteristics of the image. Radiographers are inclined to assess the quality of imaging for proper positioning, adequate exposure, patient motion blur and other defects that could potentially affect diagnosis [13]. Artefacts and processing errors are also reported for reasons radiographers reject imaging [7, 8, 14]. Technical criteria in image quality are however hard to fulfil and could lead to radiographers’ premature rejection of images that potentially provide radiologists with information of diagnostic value [12].

Several factors are reported to influence rejects of imaging among radiographers including skills and the inter-subjectivity in assessment, and attitude towards, both technical and clinical image quality criteria [12, 15]. Waaler and Hofmann [15] highlight inter-subjectivity of radiographers’ perception of image quality to be a challenge in the effort to reduce reject rate. Having higher years of work experience is reported to have some influence on how radiographers assess images, with more experienced radiographers assessing more than merely radiographic imaging criteria [16]. The difference in image quality assessment due to years of experience is also shown among radiologists. Saade et al. [17] reports of senior radiologists scoring image quality and image technique as acceptable 32% and 27% respectively more than junior radiologists. The education of the radiographers within a particular radiography subspecialty also influences assessment of image quality. Mercieca, et al. [18] reports of radiographers with no specific mammography qualifications rejecting more images than those with a specialisation.

This study is part of a larger project investigating different aspects of image quality assessments in clinical practice that may influence retake rate. The aim of this study was to survey among radiographers in medical imaging internationally to explore assessments of usability of X-ray images and the need for image retake in specific cases and compare them with results from Norway.

## Methods

A semi-structured questionnaire was distributed through the European Federation of Radiographer Societies (EFRS) research hub at the European congress of radiology (ECR) in 2020. All radiographers attending the online congress were invited to participate. Five hundred and forty-six radiographers visited the research hub in July 2020. The respondents gave written, informed consent.

The questionnaire included 30 clinical cases (5 paediatric) in addition to questions collecting demographics of the respondents. Cases were provided from the picture and archiving system of one hospital in Norway. Each case included one image and a short referral text. The questionnaire included three chest, five spine, fourteen upper-limb, and eight lower-limb X-ray cases, representing examinations shown to have high rejection rates [19]. The images were pre-assessed into three categories (Keep, could keep and reject) by the authors based on European guidelines on quality criteria for diagnostic radiographic images [1]. In the categories reject and could accept cases the images flaws were: suboptimal *positioning* (36.4%; one case of wrong extremity imaged), *collimation* (22.7%), *exposure error* (18.2%), *centering* (13.6%) and *artifacts* (9.1%).

In each case, the respondents were to choose to either keep or reject the image. When rejecting an image, the respondents were to choose from a predefined list with the following options on the main flaw of the image: positioning is suboptimal, centering is suboptimal, artefact in the image, collimation is suboptimal, or exposure error as the main reason for rejection. In addition, there was an option to comment on why the image was kept or rejected, thus providing both qualitative and quantitative data.

### Analysis

R (R Core Team, 2020) was used to perform 2-tailed chi-squared tests to explore group differences.

The written comments were analysed grouping comments on kept and rejected images and tabulating comments into the following categories. Fulfilment of image criteria, diagnostic value, radiation protection, discuss with colleague/radiologist/referrer, and other.

The Norwegian Centre for Research Data approved this study for data handling (Reference: 987929).

## Results

In total, 81 radiographers from 21 different countries responded to the survey, a response rate of 15% of participants in the EFRS research HUB. The majority of respondents were from European countries (76%), among these most respondents were working in Ireland and the UK. Demographics of the respondents are presented in Table 1.

**Table 1:** Demographics of respondents in the survey

	N (%)
**Sex**	
Male	49 (60)
Female	32 (40)
**Years of practice**	
< 5	20 (25)
05-Oct	15 (18)
> 10	46 (57)
**Country of practice**	
North European	10 (12)
East/south European	18 (22)
Ireland	23 (28)
UK	11 (14)
African	8 (10)
Oceanian	7 (9)
Other	4 (5)

All participants answered all cases and documented 1176 comments. The respondents kept on average 63% of the images, in the “Keep” category on average 84% (SD 10) of the images were kept. In the “Could keep” category on average 63% (SD 22) of the images were kept, and in the “Reject” category the average keep rate was 43% (SD 23). There were no significant difference in rate of kept images comparing years of experience, or when comparing respondents from European and non-European countries. The Chi-squared test was used to compare European radiographers in this study to radiographers and radiologist in a previous identical survey conducted in Norway [11] (Table 2). Radiographers from the UK and Ireland kept significantly more images than those from Norway in the “Could keep” and “Reject” categories. While radiographers from the rest of Europe kept significantly more images than those from Norway in all categories. There was no significant difference between radiographers in Europe and the radiologist in the survey conducted in Norway. There was no significant difference between radiographers in UK/Ireland and the rest of Europe (data not shown).

**Table 2:** Comparing the keep rate for radiographers in Europe to radiographers and radiologists from Norway using the two-tailed chi-square test with 1 degree of freedom. Data from Norway is previously published [11]

Image category	Kept—n (%)	Cases in total	Kept—n (%)	Cases in total	*p*	Kept—n (%)	Cases in total	*p*
	Radiographers UK/Ireland		Radiographers Norway			Radiologists Norway		
Keep	226 (83)	272	274 (82)	336	0.69	98 (88)	112	0.35
Could keep	226 (60)	374	214 (46)	462	< 0.001	90 (58)	154	0.75
Reject	185 (50)	374	162 (35)	462	< 0.001	72 (47)	154	0.64

	Radiographers Rest of Europe		Radiographers Norway			Radiologists Norway		
Keep	198 (88)	224	274 (82)	336	0.04	98 (88)	112	0.95
Could keep	201 (65)	308	214 (46)	462	< 0.001	90 (58)	154	0.18
Reject	147 (48)	308	162 (35)	462	< 0.001	72 (47)	154	0.9

### Reasons for rejecting an image

**Fig. 1:**
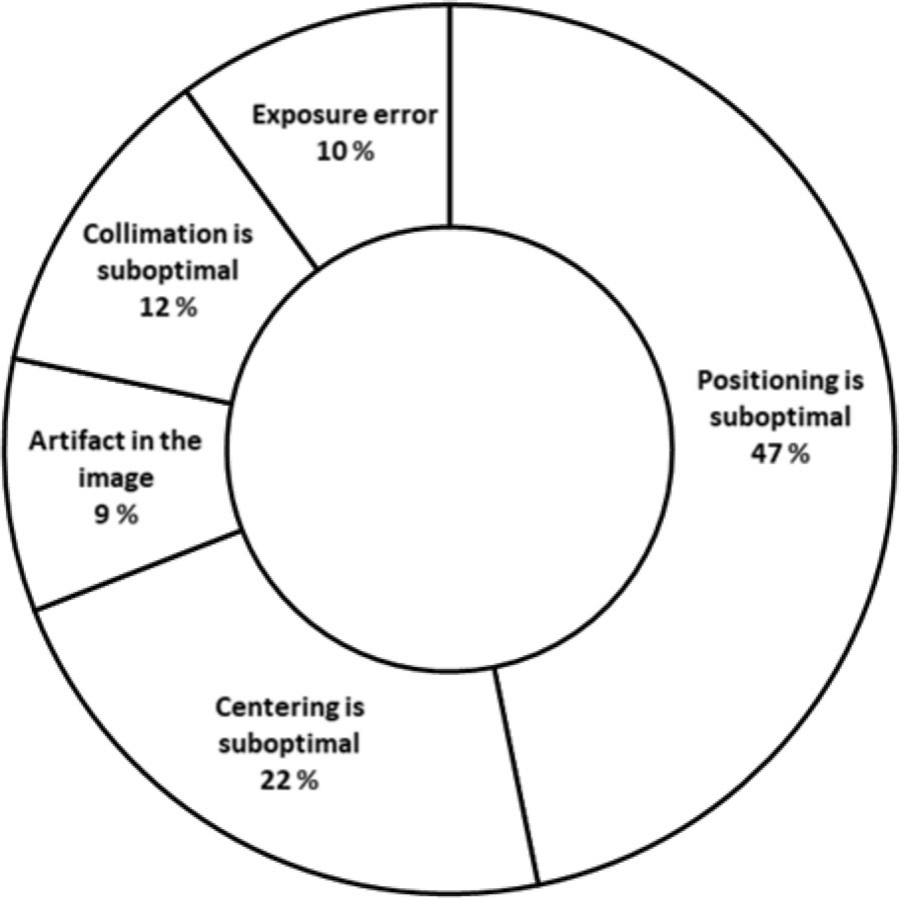
Overview of main reasons for rejection of images

Figure 1 gives an overview of the reasons given for rejecting images in all categories. Positioning is suboptimal (47%) and centering is suboptimal (22%) were the main reasons given for rejecting an image, which is a higher rate than the initial assessment where these reasons were used in 27% and 10% of images respectively.

### Comments on choice to keep or reject

Seven hundred and eleven comments were documented on kept images, while 456 comments were on rejected images; all images had comments (2–49 per case). Comments were sorted into two categories depending on whether the elaboration was written for a kept or rejected image. Table 3 presents the sub-categories in each main category.

**Table 3:** Overview of categories of comments on kept and rejected images

Keep	Image criteria (*n* = 292) Diagnostic value (*n* = 353) Radiation protection (*n* = 33) Check with radiologist (*n* = 5) Other (*n* = 28)
Reject	Image criteria (*n* = 292) Diagnostic value (*n* = 353) Supplementary imaging needed (*n* = 4) Other (*n* = 28)

For kept images, the most common comments (*n* = 353) were on how the image could have diagnostic value even though the image criteria were not fulfilled. In other cases, the elaboration was used to comment that image criteria were met (*n* = 292). In addition, the thirty-three comments discussed that radiation protection would be a reason to keep an image of low quality and five comments referred to confer with a radiologist or referring physician before deciding to keep or reject.

For rejected images, most comments were on lack of image criteria fulfilment (*n* = 439), including missing markers. Twelve comments discussed lack of diagnostic value, while four elaborations were on the possibility to keep the image if the other images in the series compensated for the information missing in the current image.

## Discussion

In this study, we found that radiographers kept on average 63% of the cases. Images pre-assessed as of low quality were rejected most often. The comments on rejected images focused on lack of image criteria fulfilment or low diagnostic quality. While comments on kept images focused on diagnostic value despite image criteria not being fulfilled or stating that image quality criteria was fulfilled in images of good quality.

Comparing radiographers in different parts of Europe to radiographers and radiologists in Norway showed that radiographers from Norway rejected a significantly higher proportion of images. There was no significant difference between radiologists from Norway and radiographers from other European countries.

### Image criteria verses diagnostic quality

The reported reasons for a rejected image in our study were attributed to technical skills or human errors. Insufficient patient positioning (47%) was the main given reason for rejection. The image criteria specify important anatomical structures that should be visible on a radiograph to aid accurate diagnosis which of these criteria fundamentally depend on accurate patient positioning [2]. Therefore, proper positioning of the patient is one important required factor in production of high-quality images of diagnostic value. Our study does however indicate from the comments that the radiographers would also consider diagnostic value in assessing whether to keep or reject an image. The radiographers’ role is to ensure that imaging of diagnostic quality is obtain and the radiologists interprets the images and provide diagnosis. Where a low quality image provides sufficient information for accurate diagnosis, the radiologist is able to answer the clinical question and thus would be unnecessary to repeat imaging [17]. However, images rejected by radiographers are mostly not reviewed by the radiologist [19]. Even though diagnostic value would mainly be the radiologist domain radiographers need to consider the images diagnostic value if an image does not fully fulfil image criteria, sometimes in dialog with the radiologists and in other cases with the support of another radiographer. The difference in rejects of images among radiographers and radiologist regarding the issue of technical versus diagnostic capability is reported. Mount [12] reports that radiographers’ focus on technical characteristics of an image can lead to premature rejection of images of diagnostic value. Evaluation on technical criteria can also be difficult to satisfy thus leading to more rejects [13]. To prevent unnecessary imaging retakes it is important that discussions to weigh technical characteristics verse diagnostic benefits between radiographers and radiologists occur [19]. Thus, training in image quality assessment is central for radiographers.

### Difference in training

Our study shows variation in rejection rates among radiographers in the included European countries. Many factors such as training or work experience as reported by several studies could have an influence [16–18]. Training of radiographers to improve the technical skills and operation of equipment is vital to reduce imaging errors [20]. McFadden et al. [21] advocated for standardisation of education and training within Europe including protocols and exposure parameters to ensure that there is continued adherence to the ALARA principle. Practical aspects in routine clinical practice are also vital to reduce technical errors. Lin et al. [7], recommends introduction of position and equipment technical guidelines on required patient positioning and adding poster reminders to radiographers to assist to reduce positioning errors and rejects. Current advancement in deep learning and artificial intelligence should possibly resolve or reduce rejects caused by technical or human errors particularly patient position when implemented [22].

### Radiation protection

Our study shows from the comments that radiation protections was also an important factor for keeping an image of lower quality. Rejected images increase radiation exposure to patients, increasing the risk of stochastic effects [4]. The risk of stochastic effects are dependent on age, sex and individual health status [5]. The accumulative dose exposure from previous radiological procedures should also be considered before rejecting and repeating the radiographic image [23]. This further highlights the importance of discussions with radiologists particularly when assessing the need for retakes in the vulnerable groups such as in paediatric imaging. Ensuring patient radiation safety and minimising exposure of ionising radiation or other harm in imaging is fundamental to healthcare in radiology [4].

### Limitations of study

The response rate was low only 15%. A low response rate reduces strength of analysis with regards to producing a representative result [24]. Due to the Covid-19 pandemic, the ECR 2020 was held virtually, and the distributed survey was displayed to visitors at an online research hub along with several other surveys. The response rate was calculated from the number of participants who visited the hub web page. There may have been visitors to the page who did not participate in any surveys and thus the rate may have been under reported. It was also not possible to prompt individuals directly or send reminders to visitors of the page. This may have influenced the number of potential participants reached, further contributing to the low response rate. Additionally, the initial assessment of image quality of the cases in the survey is based on guidelines and subjective assessment of the authors. This approach has limitations. Discussing the cases with radiologists and other radiographers could have helped to strengthen validity. The survey was presented in English only. This may have hindered some radiographers’ participation causing skewedness in number of participants from English speaking countries in Europe. Language could have further caused misunderstanding of the presented cases for non-native English-speaking participants. On the other hand, English is the language of official communication and correspondence at the ECR. The participants would thus be expected to understand English.

## Conclusion

This study shows that there is an agreement internationally on what makes a good quality X-ray image. However, the opinion on medical usefulness of images of low or poor quality compared to image criteria varies. The radiographers considered the lack of fulfilment of image criteria as a reason for rejecting an image whereas diagnostic capability was an important reason for keeping images of low quality. There seems to be a difference in image rejection rate in this survey based on whether radiographers strictly adhere to the image criteria rather than assessing diagnostic quality. Our study suggests that radiographers from Norway reject more images based on lack of fulfilment of image criteria than their international colleagues. Radiologists in Norway seems be on a similar level of rejection as radiographers internationally. In addition to regular image reject analysis and training for radiographers in image assessment, we highly recommend radiographers and radiologists to communicate on image quality in clinical practice, and for diagnostic quality to be included in image criteria assessment.

## Data Availability

The datasets used and/or analysed during the current study are available from the corresponding author on reasonable request.

## Abbreviations

ALARA: As Low As Reasonably Achievable
ECR: European congress of radiology
EFRS: European Federation of Radiographer Societies
UK: United Kingdom
